# Significant decrease of von Willebrand factor and plasminogen activator inhibitor-1 by providing supplementation with selenium and coenzyme Q10 to an elderly population with a low selenium status

**DOI:** 10.1007/s00394-020-02193-5

**Published:** 2020-02-20

**Authors:** Urban Alehagen, J. Alexander, J. Aaseth, A. Larsson, T. L. Lindahl

**Affiliations:** 1 Division of Cardiovascular Medicine, Department of Medical and Health Sciences, Linköping University, 581 85 Linköping, Sweden; 2 Norwegian Institute of Public Health, 0403 Oslo, Norway; 3 Research Department, Innlandet Hospital Trust, 2381 Brumunddal, Norway; 4 Department of Medical Sciences, Uppsala University, 751 85 Uppsala, Sweden; 5 Division of Clinical Chemistry, Department of Experimental and Clinical Medicine, Linköping University, 581 85 Linköping, Sweden

**Keywords:** Von willebrand factor, PAI-1, Intervention, Elderly, Selenium, Coenzyme Q10

## Abstract

**Purpose:**

Endothelial dysfunction and inflammation are conditions which fuel atherosclerosis and ischaemic heart disease. We have previously reported reduced cardiovascular (CV) mortality following supplementation with selenium and coenzyme Q10 to 443 elderly individuals with low selenium status (mean 67 μg/L) for 4 years. Here, we wanted to evaluate a possible association between the supplementation and the plasma concentrations of the von Willebrand factor (vWf), and the plasminogen activator inhibitor-1 (PAI-1), as they, besides other functions, are also strongly associated with endothelial function.

**Methods:**

In this sub-study, 308 individuals (active substance: 157, placebo: 151) were included. Blood samples were drawn after 6 and 36 months and vWf and PAI-1 were determined in plasma by ELISA. Changes in concentrations of the biomarkers were evaluated by the use of *T* tests, repeated measures of variance, and ANCOVA analyses.

**Results:**

The active treatment group presented a lower level of vWf after 36 months compared with the placebo group (1.08 U/mL vs. 5.10 U/mL; *p* = 0.0007). The results were validated through the repeated measures of variance evaluation. The PAI-1 levels showed an equally significant decrease in the active group (26.2 ng/mL vs. 49.2 ng/mL; *p* = 0.0002) and were also validated through repeated measures of variance evaluation.

**Conclusion:**

In this sub-study on elderly receiving selenium and coenzyme Q10, or placebo we found significantly lower levels of vWf and PAI-1 in the active treatment group as compared to the placebo group. We interpret this as a better endothelial function because of the intervention, which accords with a previous finding of reduced CV mortality.

## Introduction

Selenium is one of the trace elements found as selenoproteins in the living cells that are essential for a normal cellular function [1, 2]. The most important selenoproteins are selenoprotein P, glutathione peroxidases, and thioredoxin reductase, all of which protect against oxidative stress.

In Europe, a low selenium content of the soil results in low dietary selenium intake [3–7], and biofortification has therefore been proposed [8, 9]. In contrast, in the United States the selenium content in the soil is generally higher [10, 11].

Ubiquinone (coenzyme Q10) is a powerful antioxidant that is also needed for optimal function of all living cells [12]. An intricate interrelationship between selenium and coenzyme Q10 has been reported Xia et al., indicating that both groups are needed in order to obtain normal cellular function, and are related to each other [13]. The endogenous production of coenzyme Q10 decreases with increasing age, where the endogenous production of coenzyme Q10 in the myocardium is about half at the age of 80 compared to 20 years [14].

A trial where elderly community-living persons received a combined supplementation of selenium and coenzyme Q10 for 4 years has been published [15]. The choice of giving both selenium and coenzyme Q10 as supplementation was based on the facts that a low selenium intake had been shown in the same population, a decreased endogenous production of coenzyme Q10 was known due to the high age, and that there is an interrelationship between the two substances within the cell. One of the main results from the study was a significant reduction in cardiovascular (CV) mortality [15].

The von Willebrand factor (vWf) is a plasma glycoprotein with an important function in primary haemostasis and in coagulation as it is the major carrier for factor VIII. vWF is produced and stored in endothelial cells and platelets. It mediates the adhesion process of platelets to endothelial surfaces [16], where the vWf is anchored to the endothelium through fibres [17]. The factor has a multitude of functions, including controlling angiogenesis [18], and also acting as an acute phase reactant as the Weibel–Palade bodies release the inflammation mediators [19]. However, there have been observations indicating that an increased concentration of vWf is also a sign of vascular dysfunction [19]. Several reports have demonstrated the association between increased concentration of vWf and CV disease or myocardial infarction [20–22].

Plasminogen activator inhibitor-1 (PAI-1) has well-known antifibrinolytic effects, and is secreted from endothelial cells and hepatocytes [23, 24] and also synthesised and stored in platelets [25]. It is a serpin and exerts its antifibrinolytic effect by forming a 1:1 complex with tissue plasminogen activator upon cleavage of its “bait” peptide bond Arg_346_–Met_347_ [26]. Interesting data indicate that PAI-1 is also involved in the development of diabetes type II, and in insulin resistance [27, 28]. Also, several reports have pointed to an association between PAI-1 and ischaemia and coronary artery pathology as a higher level of PAI-1 has been observed in patients with ischaemic heart disease or myocardial infarction [29, 30]. Endothelial dysfunction of the coronary arteries could also be found in those without diabetes, but with signs of insulin resistance [31].

*Aim* We have previously reported the effect of supplementation with selenium and coenzyme Q10 on the level of inflammation as seen on biomarkers for inflammation. In this sub-study we wanted to evaluate a possible effect of the intervention on endothelial function and inflammation, applying the concentration of the biomarkers vWf and PAI-1, as markers of endothelial function.

## Methods

### Subjects

This is a sub-study of a prospective randomised double-blind placebo-controlled trial where 443 elderly community-living subjects in the age range 70–88 years were subjected to dietary supplementation of selenium and coenzyme Q10 for 4 years. From the main study, significantly reduced CV mortality, increased cardiac systolic function, and a reduced concentration of the cardiac peptide NT-proBNP could be reported as results of the intervention [15]. The subjects were given dietary supplementation of 200 mg/day of coenzyme Q10 capsules (Bio-Quinon 100 mg B.I.D, Pharma Nord, Vejle, Denmark) and 200 µg/day of organic selenium yeast tablets (SelenoPrecise 200 µg, Pharma Nord, Vejle, Denmark) (*n* = 221), or a similar placebo (*n* = 222) over 48 months, after which the intervention was finished. The study supplementation was taken in addition to regular medication if used. All study medications (active drug and placebo) not consumed were returned and counted. All participants were examined by one of three experienced cardiologists. A clinical history was recorded at inclusion, and a clinical examination was performed, including blood pressure, assessment of New York Heart Association functional class (NYHA class) as well as ECG and echocardiography. Doppler echocardiographical examinations were performed with the participant in the left lateral position. The ejection fraction (EF) readings were categorised into four classes with interclass limits placed at 30%, 40% and 50% [32, 33]. Normal systolic function was defined as EF ≥ 50%, while severely impaired systolic function was defined as EF < 30%. The inclusion started in January 2003 and finished in February 2010.

CV mortality was registered for all study participants. The mortality information was obtained from the National Board of Health and Welfare in Sweden, which registers all deaths of Swedish citizens based on death certificates or autopsy reports. Written, informed consent was obtained from all patients.

### Ethical approval

The study was approved by the Regional Ethical Committee (Forskningsetikkommmitten, Hälsouniversitetet, SE-581 85 Linköping, Sweden; No. D03-176), and conforms to the ethical guidelines of the 1975 Declaration of Helsinki. (The Medical Product Agency declined to review the study protocol since the study was not considered a trial of a medication for a certain disease but rather one of food supplement commodities that are commercially available). This study was registered at Clinicaltrials.gov and has the identifier NCT01443780. Since it was not mandatory to register at the time the study began, the study has been registered retrospectively.

### Biochemical analyses

Blood samples were collected after 6 and 36 months while the participants were resting in a supine position. Pre-chilled, EDTA vials containing plasma were used. The vials were centrifuged at 3000*g*, + 4 °C, and were then frozen at −70 °C. No sample was thawed more than once.

### Determination of the von Willebrand factor and PAI-1

Von Willebrand factor concentration was measured by ELISA with the Technozyme^®^ vWF:Ag kit and PAI-1 with the Technozyme^®^ PAI1:Ag kit. Both kits were purchased from Technoclone, Vienna, Austria. The inter assay variation was 6.0% and 5.5%, respectively according to the manufacturer.

### Statistical methods

Descriptive data are presented as percentages or mean ± SD. A Student’s unpaired two-sided *T* test was used for continuous variables and the chi-square test was used for the analysis of one discrete variable. Repeated measures of variance were used to obtain better information on the individual changes in the concentration of the biomarker analysed, compared to group mean values.

As the analysis of variance (ANOVA) algorithm can handle a slight non-Gaussian distribution, non-transformed data were applied in the repeated measures of variance evaluation. In the analysis of covariance (ANCOVA) evaluation, both transformed and non-transformed data were applied, with no significant difference in the results.

In the ANCOVA evaluation, the biomarker concentration after 42 months was used as an independent variable. In the model, adjustments were made for age, hs-CRP, biomarker concentration after 6 months, smoking, hypertension, ischaemic heart disease (IHD), diabetes, and supplementation with selenium and coenzyme Q10, and in the PAI-1 evaluation, also for Body Mass Index.

*p* values < 0.05 were considered significant, based on a two-sided evaluation. All data were analysed using standard software (Statistica v. 13.2, Dell Inc, Tulsa, OK, USA).

## Results

In this sub-study, the study population consisted of 308 individuals, of which 157 received active treatment, and 151 received placebo. The figures are based on the number of participants still alive after 36 months, and within the study and who agreed to deliver blood samples after 6 months and after 36 months.

From Table 1 it can be seen that the baseline characteristics are balanced between the two groups at inclusion and thus no significant difference could be seen between the groups (Table 1).

**Table 1:** Baseline characteristics of the study population receiving intervention of a dietary supplementation of selenium and coenzyme Q10 combined during 4 years

	Active	Placebo	*p* value
*N*	157	151	
Age years mean (SD)	76 (3)	77 (3)	0.34
Males/females *n*	79/78	72/79	
History			
Diabetes *n* (%)	32 (20.4)	29 (19.2)	0.80
Smoking *n* (%)	11 (7.0)	13 (8.6)	0.60
Hypertension *n* (%)	114 (72.6)	109 (72.2)	0.93
IHD *n* (%)	28 (17.8)	35 (23.2)	0.25
NYHA class I *n* (%)	93 (59.2)	76 (50.3)	0.12
NYHA class II *n* (%)	39 (24.8)	47 (31.1)	0.22
NYHA class III *n* (%)	24 (15.3)	26 (17.9)	0.65
NYHA class IV *n* (%)	0	0	
BMI mean (SD)	27.4 (4.07)	27.0 (4.18)	0.44
Hb < 120 g/L *n* (%)	15 (9.6)	16 (10.6)	0.76
Medications			
ACEI/ARB *n* (%)	30 (19.1)	40 (26.5)	0.12
Beta blockers *n* (%)	56 (35.7)	53 (35.1)	0.92
Diuretics *n* (%)	49 (31.2)	61 (40.4)	0.09
Statins *n* (%)	35 (22.3)	32 (21.2)	0.81
Examinations			
EF < 40% *n* (%)	12 (7.6)	11 (7.3)	0.90
Atrial fibrillation *n* (%)	9 (5.7)	12 (7.9)	0.44
s-Selenium µg/L, mean (SD)	66.6 (15.9)	67.4 (17.2)	0.56

*ACEI* ACE-inhibitors, *ARB* angiotension receptor blockers, *EF* ejection fraction, *IHD* ischemic heart disease, *NYHA* New York Heart Association functional class, *SD* standard deviation

In the study population, 61 out of 308 (19.8%) had diabetes, 223 out of 308 (72.4%) had hypertension, and 63 out of 308 (20.5%) had IHD thus they were representative of an elderly population in the community.

### Relation between the levels of the von Willebrand factor and PAI-1, and cardiovascular mortality

In order to determine whether the levels of the vWf and PAI-1 differed in those participants of the placebo group who had suffered CV mortality within 5, 10, and 12 years of follow-up, the levels of vWf and PAI-1 in the blood samples drawn after 6 months from the two groups were compared. After 5 years, 10 participants had suffered CV mortality, while 113 had not, after 10 years 42 participants had suffered CV mortality, while 81 had not, and finally after 12 years 49 participants had suffered CV mortality and 74 had not in this sub-population. Only those who received the placebo intervention, thus no active supplementation of selenium and coenzyme Q10 were included in this analysis.

After 5 years, numerically higher concentrations of vWf could be seen in the CV mortality group, although these concentrations were not significant, probably due to the small sample size (vWf: CV+: 23.7 U/mL vs CV−: 12.0 U/mL; *p* = 0.20). After 10 years the numerical difference persisted, and a borderline significance was obtained regarding the difference between the two groups (CV+: 19.6 U/mL vs CV−: 9.4; *p* = 0.05). After 12 years an even greater difference was obtained, which was now statistically significant (CV+: 22.4 U/mL vs CV−: 6.8 U/mL; *p* = 0.002).

Performing the same evaluation regarding PAI-1, the same message could be found. After 5 years of follow-up, a higher numerical value for concentration could be found, although not significant, probably due to the small sample size (CV+: 43.3 ng/mL vs CV−: 11.7 ng/mL; *p* = 0.20. After 10 years of follow-up a significant difference in concentration could be found (CV+: 50.4 ng/mL vs 22.0 ng/mL; *p* = 0.048), and with a follow-up period of 12 years, in those with a CV mortality a numerically higher level of PAI-1 could be seen (48.4 U/mL compared to 28.3 U/mL; *p* = 0.2) compared to those who did not suffer CV mortality.

Obviously, an association between the level of the biomarker and CV mortality could be seen. Therefore, the evaluation of supplementation of selenium and coenzyme Q10 and consideration of its possible association with the levels of biomarkers are of interest.

### Influence of selenium and coenzyme Q10 on the concentration of the von Willebrand factor

At the start of the evaluation, after 6 months, no difference in the level of vWf could be seen (active group: mean 4.6 U/mL vs placebo: 4.5 U/mL; *p* = 0.99). However, after 36 months a significantly higher level of vWf could be seen in the placebo group compared with the active treatment group (5.1 U/mL vs 1.1 U/mL; *p* = 0.0007).

Analysing the placebo group during the evaluation period, no difference in level could be demonstrated (6 months: mean 4.5 U/mL vs. 36 months: 5.1 U/mL; *p* = 0.78). However, in the active treatment group a significant decrease in the level of vWf could be seen (4.6 U/mL vs 1.1 U/mL; *p* = 0.03).

**Fig. 1:**
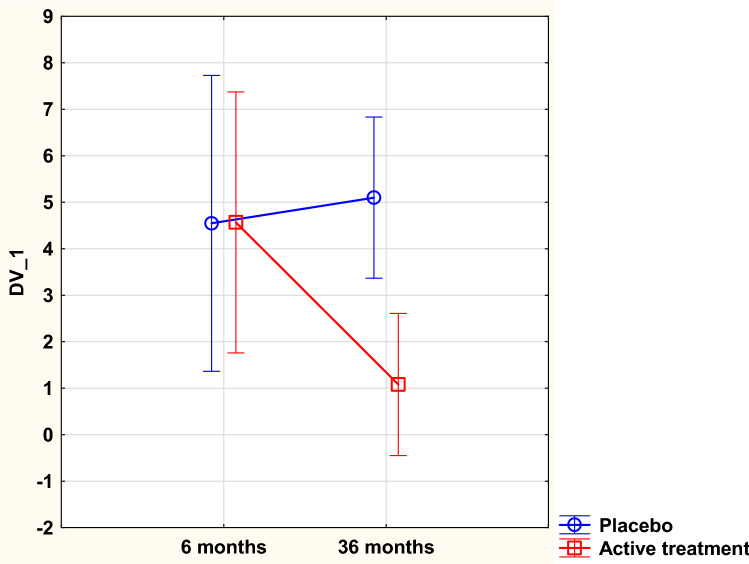
Concentration of the von Willebrand factor after 6 and 36 months in the selenium and coenzyme Q10 treatment group compared to the placebo group in the study population. Evaluation performed by the use of repeated measures of variance methodology. Current effect: *F*(1,174) = 5.19; *p* = 0.024. Vertical bars denote 0.95 confidence intervals. Blue curve: placebo; red curve: active treatment group. Bars indicate ± 95% CI

Applying the repeated measure of variance methodology in order to trace changes between the two time-points with a focus on individual changes, significant differences could also be demonstrated [*F*(1,174) = 5.20; *p* = 0.02] (Fig. 1).

To adjust for other variates that could possibly influence the levels of vWf, an ANCOVA evaluation was performed (Table 2). From this evaluation, a significant decrease in vWf concentration could be seen in the group receiving supplementation with selenium and coenzyme Q10 (*p* = 0.006).

**Table 2:** Analysis of covariance using von Willebrand factor after 36 months as dependent variable

Effects	Sum of squares	*F*	*p*
Intercept	14.2	0.23	0.64
Age	13.4	0.21	0.64
HsCRP	9.9	0.16	0.69
vWf 6 months	3829.2	61.3	< 0.0001
Smoker	8.3	0.13	0.72
Hypertension	3.5	0.06	0.81
Diabetes	4.4	0.07	0.79
IHD	24.1	0.39	0.54
Active treatment	501.6	8.03	0.006
Error	4621.6		

*HsCRP* high sensitivity assay of CRP, *IHD* ischemic heart disease, *vWf* von Willebrand factor

### Influence of selenium and coenzyme Q10 on the concentration of PAI-1

At the start of the evaluation, no difference in the concentration of PAI-1 between the active treatment group and the placebo group could be found (45.6 ng/mL vs 48.5 ng/mL; *p* = 0.79). However, after 36 months a significantly lower concentration of PAI-1 could be seen in the active treatment group compared with the placebo group (mean 26.2 ng/mL vs. 49.2 ng/mL; *p* = 0.0002).

In the placebo group, no significant difference in concentration during the evaluation period could be demonstrated (mean 48.5 ng/mL vs. 49.2 ng/mL; *p* = 0.95), whereas in the active treatment group, a significantly lower concentration was found (45.6 ng/mL vs. 26.2 ng/mL; *p* = 0.01).

**Fig. 2:**
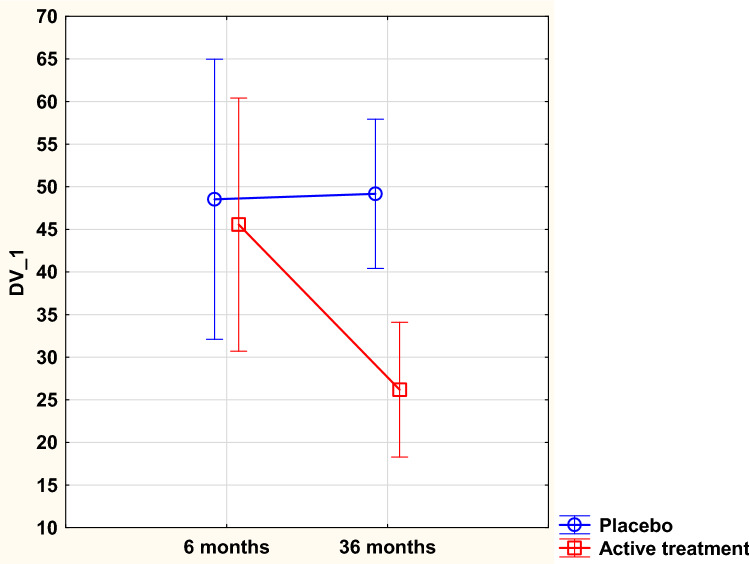
Concentration of the plasminogen activator inhibitor-1 after 6 and 36 months in the selenium and coenzyme Q10 treatment group compared to the placebo group in the study population. Evaluation performed by the use of repeated measures of variance methodology. Current effect: *F*(1,176) = 4.38; *p* = 0.038. Vertical bars denote 0.95 confidence intervals. Blue curve: placebo; red curve: active treatment group. Bars indicate ± 95% CI

Applying repeated measure of variance regarding the change in concentration in PAI-1, a significant difference between the active treatment group and the placebo group could be demonstrated [*F*(1,176) = 4.4;* p* = 0.037] (Fig. 2). Performing an ANCOVA evaluation, a significantly lower concentration (*p* = 0.0005) could be seen in those supplemented with selenium and coenzyme Q10, also after adjusting for variates that might influence the concentration of PAI-1 (Table 3).

**Table 3:** Analysis of covariance using PAI-1 after 36 months as dependent variable

Effects	Sum of squares	*F*	*p*
Intercept	37.8	0.07	0.80
Age	48.8	0.09	0.77
HsCRP	308.4	0.55	0.46
PAI-1, 6 months	23,806.4	42.6	< 0.0001
BMI	1597.4	2.9	0.09
Smoker	412.1	0.74	0.39
Hypertension	126.3	0.23	0.64
Diabetes	160.8	0.29	0.53
IHD	48.6	0.09	0.77
Active treatment	7857.8	14.1	0.0003
Error	41,326.8		

*BMI* Body Mass Index, *HsCRP* high sensitivity assay of CRP, *IHD* ischemic heart disease

## Discussion

In this sub-study of the KiSel-10 study, where supplementation with selenium and coenzyme Q10 or placebo were provided to elderly community-living participants during 4 years, endothelial function and inflammation were evaluated as reflected by the levels of vWf and PAI-1. We have previously reported that the population was selenium deficient with a mean pre-intervention selenium serum concentration was 67.1 μg/L (SD 16.8) (equivalent to a daily intake of 35 μg) compared to an adequate status of ≥ 100 μg/L [34] and 210 μg/L (SD59.4) post-intervention [35]. After the intervention no participant exceeded a selenium concentration of 230 μg/L.

An important aspect to discuss is if this supplementation dose is associated with increased risk. It is well known that high intakes of selenium may cause toxicity, i.a. in the liver. Therefore, EU Scientific Committee on Food/ European Food Safety Authority established an upper tolerable intake level (UL) for selenium of 300 μg/day (EFSA 2006) [36]. The Intake level of Se in our study, consisting of a supplementation level of 200 µg/day on top of a dietary intake of much less than 100 µg/day (in the studied population an estimated daily selenium intake of 35 µg/day was demonstrated), was altogether far less than the EFSA UL of 300 µg. Also, the plasma concentration obtained following supplementation, i.e. ≤ 230 µg/L, was below those previously reported associated with adversity [34].

More recently, Rayman et al. reported results from an intervention study in Denmark consisting of 491 individuals [37]. Supplementation levels of 100 and 200 µg/day on top of dietary intake provided no evidence of increased risk of mortality; there were, however, wide confidence intervals. The authors claim that an increased mortality risk could be seen in the group supplemented with 300 μg/day. However, from the publication the relevant graphs indicate a confidence interval passing the neutrality line after 5 and 10 years and reaches the neutrality line at 15 years of follow-up. The point estimate showing increased mortality should, therefore, be interpreted with great caution according to standard statistical routines. Anyway, this study supports the notion that supplementation up to a level of 200 μg/day in a population with low selenium status is safe.

From the literature, we know that the endogenous production of coenzyme Q10 declines to about half at the age of 80 years old, as compared to the level produced at the age of 20 [14]. As the Q10 and the selenoenzyme thioredoxin reductase work in concert reducing Q10 to ubiquinol an important lipidsoluble antioxidant in the cell, this project supplemented both substances.

We observed that the concentration of both vWf and PAI-1 were significantly lower after 36 months of intervention in the group receiving selenium and coenzyme Q10, compared with those on placebo. This could be seen both in the group mean evaluations and by use of the repeated measures of variance methodology which focuses on individual changes.

As many other clinical variables could influence the level of the biomarkers, ANCOVA evaluations were also performed, in which adjustments for the covariates were made, but still with significant differences between the two groups in levels of the biomarkers.

We have previously observed indications of decreased inflammatory activity accompanying supplementation with selenium and coenzyme Q10, as registered by using six different biomarkers for inflammation [38, 39]. Our present findings regarding the concentrations of vWf and PAI-1 are therefore interesting as inflammation and endothelial dysfunction are closely interrelated [40].

However, as the level of vWf is also associated with other conditions such as age and blood type [41], it is important to determine the reason for the increased level by use of another biomarker for inflammation/ endothelial function—in this study, the PAI-1.

In previous studies, vWf was associated with inflammation [42] and a biomarker for vascular damage, which is why it is now also regarded as a marker for endothelial dysfunction [43].

For the second biomarker used in this study, PAI-1, reports in the literature indicate that the level of PAI-1 is associated with inflammation [44]. PAI-1 is also closely associated with endothelial dysfunction, as shown in different conditions [45], including in CV ischaemic disease and myocardial infarction [29].

That supplementation of selenium and coenzyme Q10 could influence the levels of both vWf and PAI-1, as has been shown in this study, is not surprising, even if to the best of our knowledge this is the first time that this has been demonstrated in a patient population through dietary supplementation of selenium and coenzyme Q10. Our group has recently besides effects on inflammation [38, 39], reported reduced oxidative stress following intervention with selenium and coenzyme Q10 [46], and also changes in expression of mRNA [47] central to inflammation and endothelial function. Therefore, it is not surprising that the intervention also had effect on endothelial function.

Two of the most important selenoproteins, thioredoxin reductase and glutathione peroxidase, have important roles in endothelial cell physiology [48, 49]. As a result, these enzymes regulate both the inflammatory and atherogenic processes. Thus, Stupin et al. in a rat model, observed that selenium deficiency may result in impaired vascular function and increased oxidative stress [50]. The same message could be obtained from human endothelial cells where, in sepsis, a low selenium level results in increased oxidative stress and inflammation [51]. These results concur with the finding in the present study that in those with a selenium deficiency, a positive effect could be obtained by supplementation with selenium and coenzyme Q10 combined.

The participants suffering from CV mortality after 12 years of follow-up of the placebo group exhibited a higher level of the two biomarkers at 6 months, indicating that the biomarker level represents a risk variable for CV mortality, which is interpreted as the end stage of endothelial dysfunction with inflammation. As the sample size in the present sub-study was small, the differences between the level of the biomarkers in those who suffered CV mortality after 5 and 10 years of follow-up, and the level in those without mortality did not reach statistical significance, even if the actual figures showed a similar difference.

However, the present study demonstrates that supplementation with selenium and coenzyme Q10 to elderly persons with deficiency of the substances affects the endothelial function in a positive way, which adds to the knowledge on the mechanisms behind the positive clinical effects reported earlier.

## Limitations

The presented study has included participants in a relatively narrow age stratum. It is therefore difficult to extrapolate the results into other age groups. However, one could assume that the mechanisms described above also exist in other age strata.

Also, the included study sample is relatively small, which is why the confidence intervals obtained are wide. However, in spite of the reduced size, significant results could be presented in most of the evaluations.

Finally, all included participants were Caucasians that also had a relatively low selenium status; thus, the results do not necessarily apply to other populations, e.g. a selenium replete population.

## Conclusion

The vWf, and PAI-1 act as biomarkers for endothelial function, and inflammation, besides having other different functions. In this sub-study from a 4-year intervention trial of selenium and coenzyme Q10 with elderly community-living persons with a low selenium status, the concentration of the two biomarkers was evaluated and compared between the active treatment group, and the placebo group.

Significantly lower levels of both biomarkers could be demonstrated in the supplemented group after 36 months of intervention. As we have previously reported, signs of less inflammation and an optimized endothelial function might be another of the mechanisms explaining the clinical results from the main study where reduced CV mortality could be reported.

As this is a small study, more research in the field is needed in order to better understand the positive effects of supplementation on individuals with a deficiency of selenium and coenzyme Q10.

## Abbreviations

ACEI: ACE- inhibitors
ANCOVA: Analysis of covariance
ANOVA: Analysis of variance
ARB: Angiotension receptor blockers
EF: Ejection fraction
ECG: Electrocardiogram
Hs-CRP: High sensitivity analysis of C-reactive protein
IHD: Ischemic heart disease
NYHA class: New York Heart Association functional class
PAI-1: Plasminogen activator inhibitor-1
SD: Standard deviation
vWf: Von Willebrand factor
